# Choline Plasmalogens Isolated from Swine Liver Inhibit Hepatoma Cell Proliferation Associated with Caveolin-1/Akt Signaling

**DOI:** 10.1371/journal.pone.0077387

**Published:** 2013-10-15

**Authors:** Yaoyao Zhan, Liang Wang, Jing Liu, Keli Ma, Cuiping Liu, Yuan Zhang, Wei Zou

**Affiliations:** 1 College of Life Science, Liaoning Normal University, Dalian, Liaoning, P. R. China; 2 Regenerative Medicine Center, the First Affiliated Hospital of Dalian Medical University, Dalian, Liaoning, P.R.China; 3 College of Fisheries and Life Science, Dalian Ocean University, Dalian, Liaoning, P. R. China; 4 Department of Biochemistry and Molecular Biology, Dalian medical University, Dalian, Liaoning, P. R. China; 5 Key Laboratory of Molecular Enzymology, the Ministration of Education, Jilin University, Changchun, Jilin, P. R. China; Wageningen UR Livestock Research, Netherlands

## Abstract

Plasmalogens play multiple roles in the structures of biological membranes, cell membrane lipid homeostasis and human diseases. We report the isolation and identification of choline plasmalogens (ChoPlas) from swine liver by high performance thin layer chromatography (HPTLC) and high performance liquid chromatography (HPLC)/MS. The growth and viability of hepatoma cells (CBRH7919, HepG2 and SMMC7721) was determined following ChoPlas treatment comparing with that of human normal immortal cell lines (HL7702). Result indicated that ChoPlas inhibited hepatoma cell proliferation with an optimal concentration and time of 25 μmol/L and 24 h. To better understand the mechanism of the ChoPlas-induced inhibition of hepatoma cell proliferation, Caveolin-1 and PI3K/Akt pathway signals, including total Akt, phospho-Akt(pAkt) and Bcl-2 expression in CBRH7919 cells, were determined by western blot. ChoPlas treatment increased Caveolin-1 expression and reduced the expression of phospho-Akt (pAkt) and Bcl-2, downstream targets of the PI3K/Akt pathway. Further cell cycle analysis showed that ChoPlas treatment induced G_1_ and G_1_/S phase transition cell cycle arrest. The expression of essential cell cycle regulatory proteins involved in the G_1_ and G_1_/S phase transitions, cyclin D, CDK4, cyclin E and CDK2, were also analyzed by western blot. ChoPlas reduced CDK4, cyclin E and CDK2 expression. Taken together, the results indicate that swine liver-derived natural ChoPlas inhibits hepatoma cell proliferation associated with Caveolin-1 and PI3K/Akt signals.

## Introduction

Plasmalgens are a unique subset of phospholipids in which the *sn-1*carbon of the glycerol backbone contains a vinyl ether–linked (a *cis* double bond adjacent to an ether bond) long chain hydrocarbon instead of the typical ester-linked fatty acid. In plasmalogens, the aliphatic moieties at the sn-1 position consist of C16:0 (palmitic acid), C18:0 (stearic acid) or 18:1 (oleic acid) carbon chains, whereas the sn-2 position is occupied by polyunsaturated fatty acids (PUFA) and the head group is usually either ethanolamine (ethanolamine plasmalogens, EtnPlas) or choline (choline plasmalogens, ChoPlas) [1].

These structural and compositional features provide novel properties to plasmalogens and although they represent up to 20 % of the total phospholipid mass in humans, their physiological roles have been challenging to identify and are likely particular to different tissues, metabolic processes and developmental stages[2]. Plasmalogens are enriched in brain (90% of EtnPlas), kidney and lung tissue, as well as skeletal and cardiac muscle. Mature spermatozoa contain a high proportion of both PlsEtn and PlsCho. The lowest amounts of plasmalogen are found within the liver, possibly owing to their synthesis in the liver and subsequent transport by lipoproteins to other tissues [3].

Plasmalogens have been found to serve as endogenous antioxidants, mediators of membrane structure and dynamics, and storage for polyunsaturated fatty acids and lipid mediators[1].They also play important roles in disease states including Zellweger syndrome[3], rhizomelic chondrodysplasia punctate (RCDP)[4], Alzheimer’s disease (AD) [5,6], Niemann-pick type C (NPC) [7], Down syndrome (DS) [8], neuronal ceroid lipofuscinosis (NCL) [9] and retinitis pigmentosa (RP) [10]. Additionally, Zoeller et al. showed that increasing plasmalogen levels protected human endothelia cells during hypoxia[11].

It had been demonstrated that plasmalogens are involved in HDL-mediated cholesterol efflux in plasmalogen-deficient cells[12]. Recent studies showed that selective membrane plasmalogen enhancement was related to altered cellular cholesterol processing *in vitro*[13]. Since increased cholesterol levels are commonly found in cancers [14] and cell membrane plasmalogen levels have been associated with cancer [15], plasmalogens could potentially be involved in cancer cell proliferation.

Caveolae are small, plasma membrane invaginations that contain high levels of glycosphingolipids and cholesterol. Caveolin-1(Cav-1), a 21 kDa scaffold protein, serves as a specific marker for caveolae and is also associated with enhanced cholesterol efflux [16]. Our previous studies showed that Cav-1, as a tumor regulator, is involved in cell proliferation, transformation and apoptosis of breast cancer and hepatoma [17,18]. Lisapti and other groups have demonstrated that a variety of signaling components are highly enriched in caveolae, including low molecular weight heterotrimeric G proteins, Src family kinases, EGF receptors, PDGF receptors, endothelin receptors, the phosphotyrosine phosphatase syp, Grb2, MAP kinase, protein kinase C and the p85 subunit of PI3K [19-23]. Therefore, we hypothesized that exogenous plasmalogens could interact with Cav-1 directly or indirectly to effect proliferation and growth in many kinds of cells.

In the present study, the role of exogenous natural plasmalogen in cancer cell proliferation and whether there is a direct or indirect interaction between exogenous natural plasmalogen and caveolin-1 were investigated. We also examined the signaling pathways through which exogenous natural plasmalogen influences cancer cell proliferation. ChoPlas, isolated and purified from swine liver, was used to treat hepatoma cells. Cell growth, viability, caveolin-1 expression, PI3K/Akt pathway related signals and cell cycle markers were determined and analyzed. The data provide novel insights into the role of exogenous natural plasmelogens and the basic mechanisms by which they alter cancer cell proliferation.

## Materials and Methods

### Cell lines and reagents

Rat hepatoma cell line CBRH-7919, human hepatoma cell line HepG2 and SMMC 7721, human normal hepatocytic cell line HL7702 and Chang liver were obtained from the Cell Bank of the Institute of Biochemistry and Cell Biology, Chinese Academy of Science (Shanghai,China). CyclinD1/CDK4, CyclinE /CDK2, Caveolin1, Akt/ phospho-Akt (Thr^308^, monoclonal) and Bcl-2 antibodies were from Santa Cruz Biotech, U.S.A. Sheep IgG-HRP directed against rat/rabbit was from New England Biolabs, U.S.A. Chemiluminesence (ECL) reagents were from Amersham Biosciences Corp., U.S.A. MTT and Trypsin were from Bo-De Biotechnology Co., Wuhan, China. DMEM and RPMI-1640 were purchased from Gibco, U.S.A. Newborn calf serum was from Tian-E Biotechnology Co., Lushun District, Dalian, China. Authentic phosphatidylcholine (PC) obtained from bovine heart was purchased from Sigma, U.S.A. 1-O-1'-(Z)-Octadecenyl-2-Arachidonoyl- sn-Glycero-3-Phosphocholine (18:0/20:4-ChoPlas) and 1-O-1'-(Z)-Octadecenyl-2- Docosahexaenoyl-sn-Glycero-3-Phosphocholine (18:0/22:6) were purchased from Avanti Polar Lipids, U.S.A.

A Waters 2695 HPLC system equipped with a Waters 2487 UV detector and Masslynx 4.0 assay software was used (Waters, Marseilles, USA). HPLC-grade solvents were purchased from Fischer Scientific Co., U.S.A. Reagent-grade chemicals and solvents were all purchased from China National Medicines Corporation Ltd., Beijing, China.

**Figure 1:**
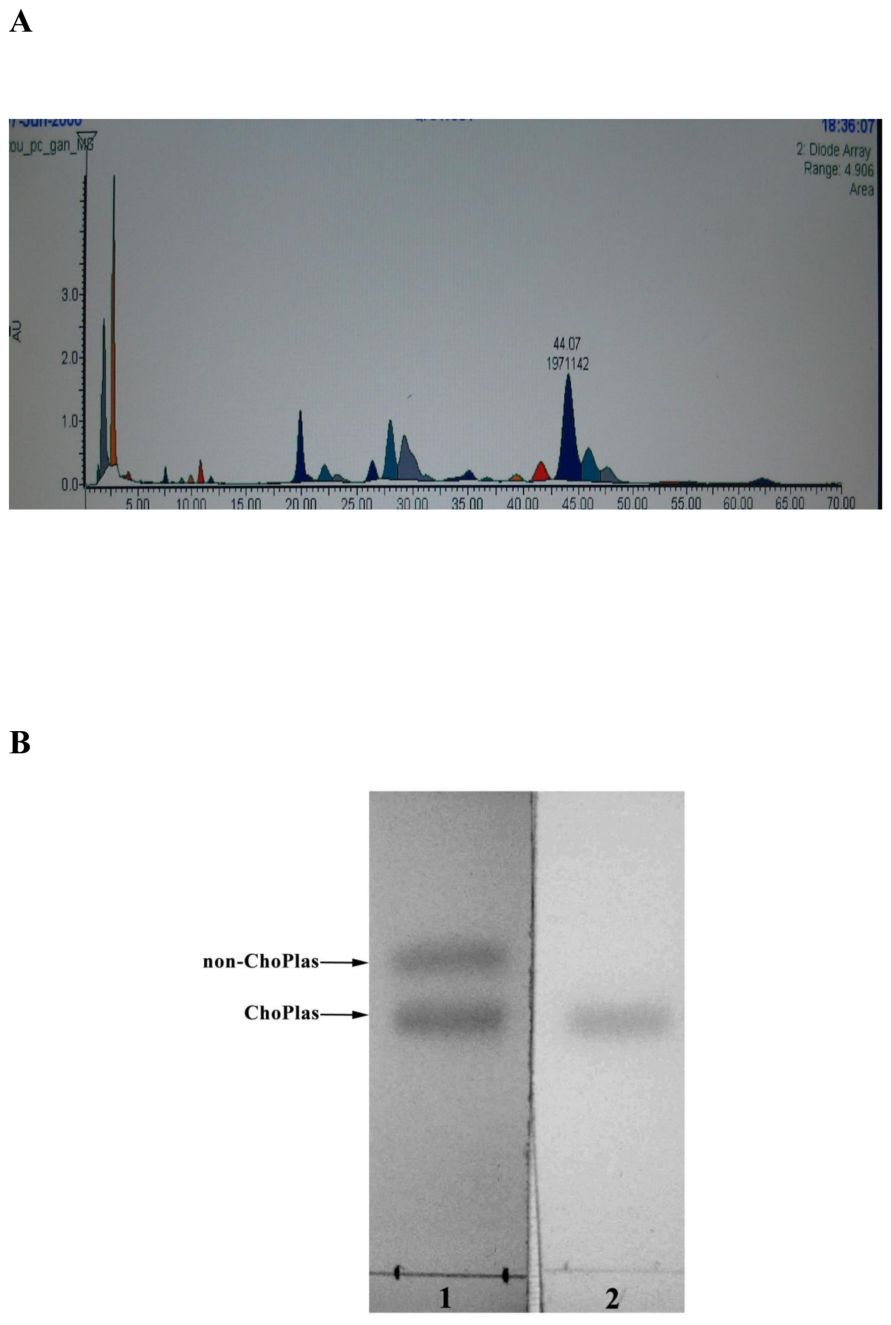
Purification and Identification of ChoPlas. **A**, **Chromatogram of PC compositions**. Kromasil C18 column; mobile phase, methanol-hexane-0.05 mol/L ammonium acetate-glycerol (V/V); column temperature, 35°C; injection, 20 μL; detection wavelength, 206 nm; flow rate, 1.0 ml/min; **B**, **High** **Performance** **Thin** **Layer** **Chromatogram** **of** **ChoPlas**. ChoPlas separated by HPLC was applied to the TLC plate and developed with chloroform/methanol/water (60:25:4, v/v, plate 1 bands were visualized by I_2_ vapor and plate 2 bands were visualized by 5 nmol/L HgCl.

### PC and ChoPlas isolation

Total lipids were extracted from swine liver according to the method outlined by Floch [20]. PC was purified on an alumina column using 90 % ethanol as the elution solvent at room temperature with a flow rate of 2.5 ml/min [21]. Purified PC was applied to the HPLC system and ChoPlas was collected and stored at -20°C for later molecular structure analysis and cellular function experiments. ChoPlas was dissolved in the medium with 50% ethanol as stocked solution before use.

### HPLC system and TLC detection

A C18 ODS HPLC column was used (4.6mm×200mm，5μm, Waters). Mobile phase A was an 85 % (v/v) methanol solution containing 6 % hexane (v/v), 8 % (v/v) ammonium acetate and 0.6 % glycerol (v/v). Separation using 22:6 ChoPlas as the internal parameter was achieved within 75 min with a flow rate of 1.00 mL/min and a column temperature of 35°C. Samples were detected at 206 nm. In order to corroborate HPLC results, purified ChoPlas was applied to a thin-layer chromatography (TLC) plate and exposed to HgCl and I_2_ vapor for 8 min and then developed in a chloroform/methanol/water mixture (60:25:4, v/v)[21].

**Figure 2:**
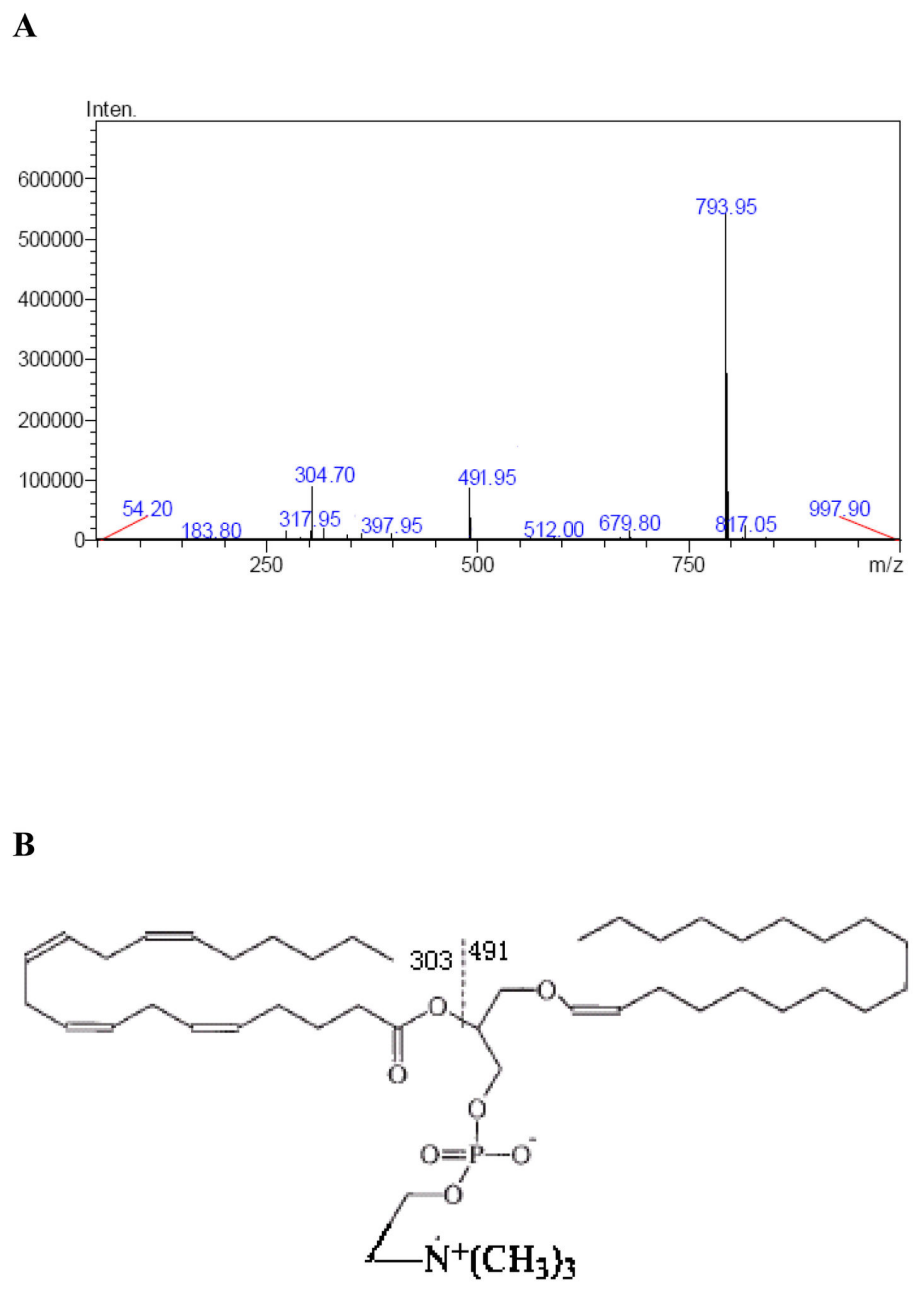
Structure Identification of ChoPlas. **A**, **Molecular characterization of ChoPlas on the MS/MS spectrum**. The mass spectrum of swine liver ChoPlas in positive mode. Elution was performed as in Figure 1. Observed ions correspond to [M+H]+ (m/z 793.95) and ions resulting from fragmentation at the sites indicated in the structure formula of the [M+H]+ anion; **B**, **Structure** **of** **the** **identified** **ChoPlas**.

### ChoPlas molecular structure identification

An HPLC-MS/MS system was used for the determination of ChoPlas. A Quattro Micro tandem mass spectrometer (Waters, U.S.A.) was used in positive electrospray ionization mode. The sheath gas was nitrogen, the collision gas was argon, the cell pressure was 25 V, the capillary temperature was 350°C, the ion source temperature was 120°C and the spray voltage was maintained at 3.0 kV.

### Cell culture

The cells lines were cultured in DMEM or RPMI-1640 medium, supplemented with 10 % FBS, 100 U/ml penicillin and 0.1 mg/ml streptomycin in a humidified incubator at 37°C under a 5 % CO_2_ atmosphere and were prepared for cell viability and western bolt analysis upon reaching sub-confluence (80–90 %).

### Cell viability assay with MTT

Cells proliferation was analyzed using 3-(4,5-dimethyl-2-thiazolyl)-2,5- diphenyl- 2H-tetrazolium bromide (MTT) . Briefly, cells were seeded in 96-well plates at a density of 1×10^4^ cells/well for 72 h in 100 μL of ordinary medium and then incubated in various concentrations of ChoPlas, which made by the stock solution for 24 or 48 h. MTT was then added to the medium and the cells were left to incubate for 4 h. The resultant insoluble formazan product was dissolved in dimethylsulfoxide. The optical density of each well was measured with a microplate reader at 490 nm. Control cells cultured without ChoPlas were defined as 100 % viable.

### Cell-cycle analysis

CBRH-7919 hepatoma cells were seeded in six-well plates and incubated with 25 μmol/L of ChoPlas for 24 h. Adherent cells were washed with PBS and 300 μL of trypsin was applied for 5 min at room temperature to collect the cells. After centrifugation at 350×g for 5 min at 4°C, the cell pellet was obtained and resuspended in 1 ml of cold 70 % ethanol at 4°C for 12 h. The cell pellet was collected again by centrifugation at 350×g for 5 min at 4°C. 1 ml of propidium iodide (PI) stain solution (20 μg/ml of PI and 8 μg/ml of DNase-free RNase) was added to the samples, which were then analyzed by flow cytometry (BD FACS Canto Trade Mark, Franklin Lakes, NJ). Mod Fit LT 3.0 software was used for data analysis.

### Western blot analysis

Total cell protein was prepared by the method described by Cui et al [22]. Cells were harvested and rinsed once with ice-cold PBS. 0.5 ml ice-cold cell lysis buffer at pH 7.5 (20 mM Tris, 150 mM NaCl, 1 mM EDTA, 1% Triton X-100, 2.5 mM sodium pyrophosphate, 1 mM glycerophosphate, 1 mM sodium orthovanadate, 1 μg/ml leupeptin, and 1 mM PMSF added directly before use) was added to each well. Cells were then scraped, transferred to Eppendorf tubes kept on ice and sonicated four times (5 s each time). The mixture was centrifuged for 10 min at 4°C and the supernatant was saved as lysate for later use. 50 μg of total protein from the lysate was separated by SDS-PAGE (10% polyacrylamide gel containing 0.1% sodium dodecyl sulfate) [23] and then transferred to PVDF membranes by electrophoretic blotting [24]. Membranes were probed with specific antibodies directed against Cavelin-1 and CyclinD1/CDK4 and CyclinE/CDK2, two signaling complexes involved in the PI3K/Akt pathway [23]. Protein bands were treated with goat anti-rabbit IgG-HRP (1:2000) and visualized by ECL using the procedure recommended by the manufacturer (Amersham Corp.). Film was exposed to the membrane for times ranging from 10 to 120 s to avoid overexposure and then was developed. Films were scanned and relative band intensities were quantified using Image J software with the control arbitrarily set as 100 %.

### Statistical analysis

Data are expressed as mean values. Differences between groups were analyzed using the Student’s t-test.

## Results

### Isolation and identification of ChoPlas

PC was purified from total lipids using alumina column chromatography. Purified PC was applied to an HPLC system to separate ChoPlas. Nineteen peaks were obtained after 10 min (Figure 1A). Peak No. 4 was identified as ChoPlas by comparison with a pure ChoPlas standard. A two-point UV-monitoring method was employed to ensure that peak No. 4 primarily contained one kind of molecule (data not shown). To further confirm the HPLC result, an optimized high performance thin layer chromatography (HPTLC) method was used for ChoPlas separation (Figure 1B)[21]. All observed results showed that ChoPlas composition was sufficient in different aspects as indicated by HPLC and HPTLC.

**Figure 3:**
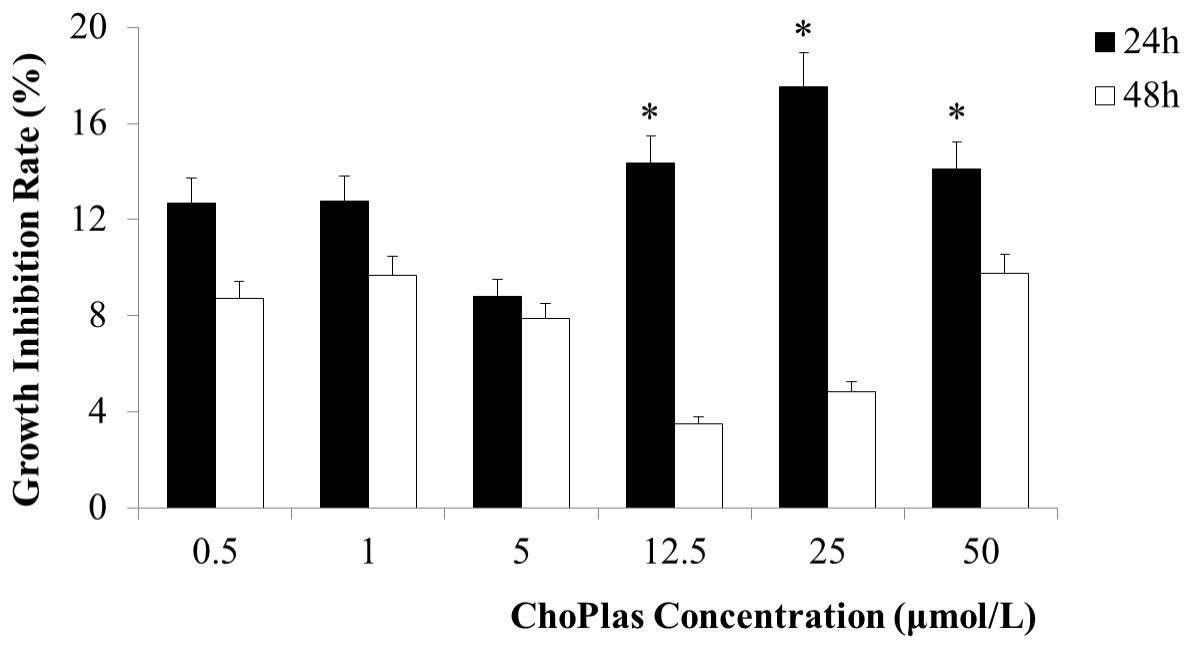
Average inhibition rate following treatment with ChoPlas as determined by MTT assay (24h and 48h). CBRH-7919 cells (1×10^4^ cells/well) were seeded into a 96 well plate with medium supplemented with 20 % FBS and incubated for 24 h. Cells were then treated with 25 μmol/L of ChoPlas for 24 and 48 h. Viable cells were stained with MTT and the resulting formazan crystals were dissolved in DMSO. Absorbances at 490 nm were measured with a multiscan plate reader. Data are presented as the mean±S.D (n=3).

### Identification of ChoPlas structure

HPLC-MS/MS is sufficiently sensitive to identify the structure of small unstable organic molecules. Peak No. 4 was collected and analyzed using a Quattro Micro tandem mass spectrometer. Under the conditions outlined above, ChoPlas molecular information was obtained (Figure 2). A full-scan mass spectra in positive ion mode of swine liver ChoPlas is shown in Figure 2A. Ions present in the figure represent the [M + H] + of ChoPlas. The fragment ions observed in the CID spectrum of m/z 794 include m/z 304 and 492 which correspond to 18:0/20:4-ChoPlas.

**Figure 4:**
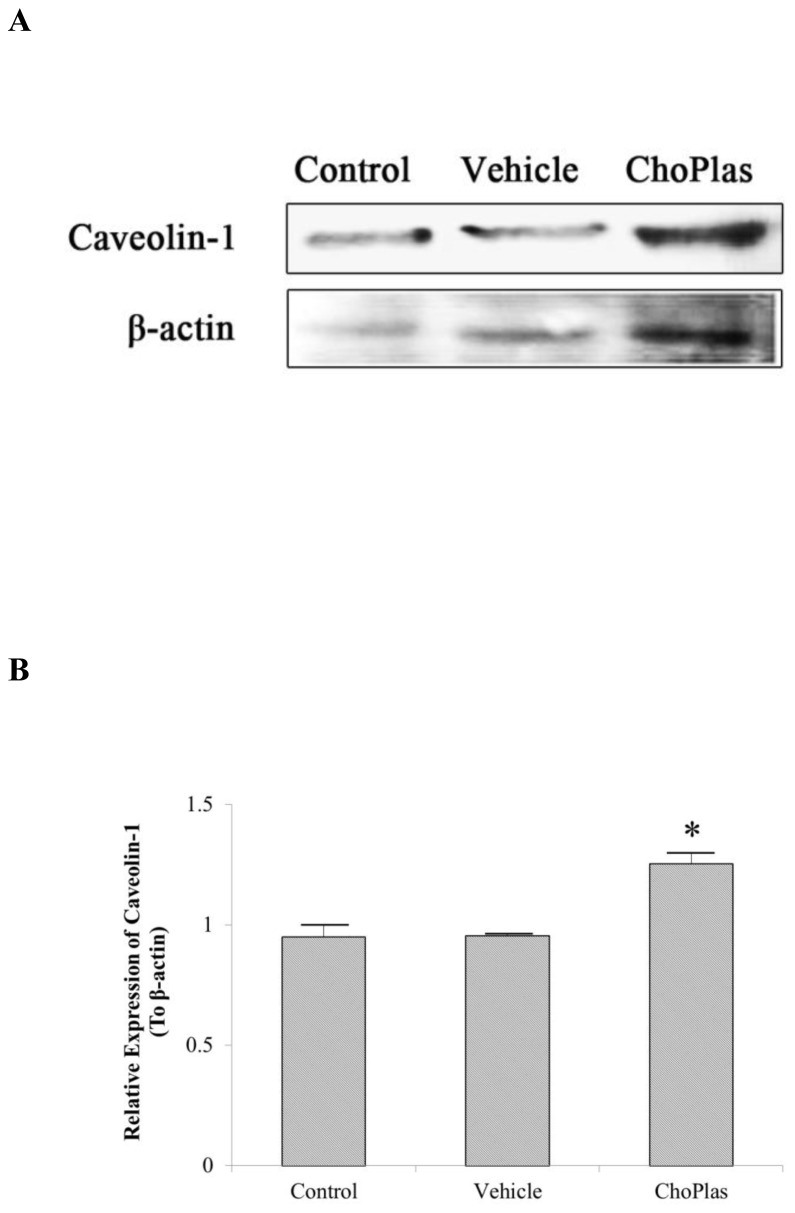
Effect of ChoPlas on Caveolin-1. CBRH7919 cells were treated with 25 μmol/L ChoPlas for 24 h and then harvested. (A)Total protein was extracted and then blotted with caveolin-1antibody; (B) Graph depicting the semi-quantization of caveolin-1expression computed using Image J software. Data are presented as the mean±S.D(n=3).

### Effect of ChoPlas on cell growth

To investigate the effect of exogenous natural ChoPlas on hepatoma cell proliferation, the impact of various ChoPlas treatments on CBRH-7919 hepatoma cells was determined by measuring MTT absorbance at 490 nm. Figure 3 shows the effect of different ChoPlas treatments on the proliferation of CBRH-7919 cells. ChoPlas inhibited hepatoma cell proliferation with a range of inhibition from 13.51 % to 17.53 % compared with control. The optimal concentration and time was 25 μmol/L of ChoPlas for 24 h. To observe whether ChoPlas inhibit cell proliferation of cancer cells not normal cells, we took not only two of human hepatoma cell lines, HepG2 and SMMC7721, but two of non-hepatoma cell lines, HL7702 and Chang’s liver cells as control. We found that it could affect on hepatoma cell lines, however, the inhibition rate on normal cell was very low (See Figure S1).

**Figure 5:**
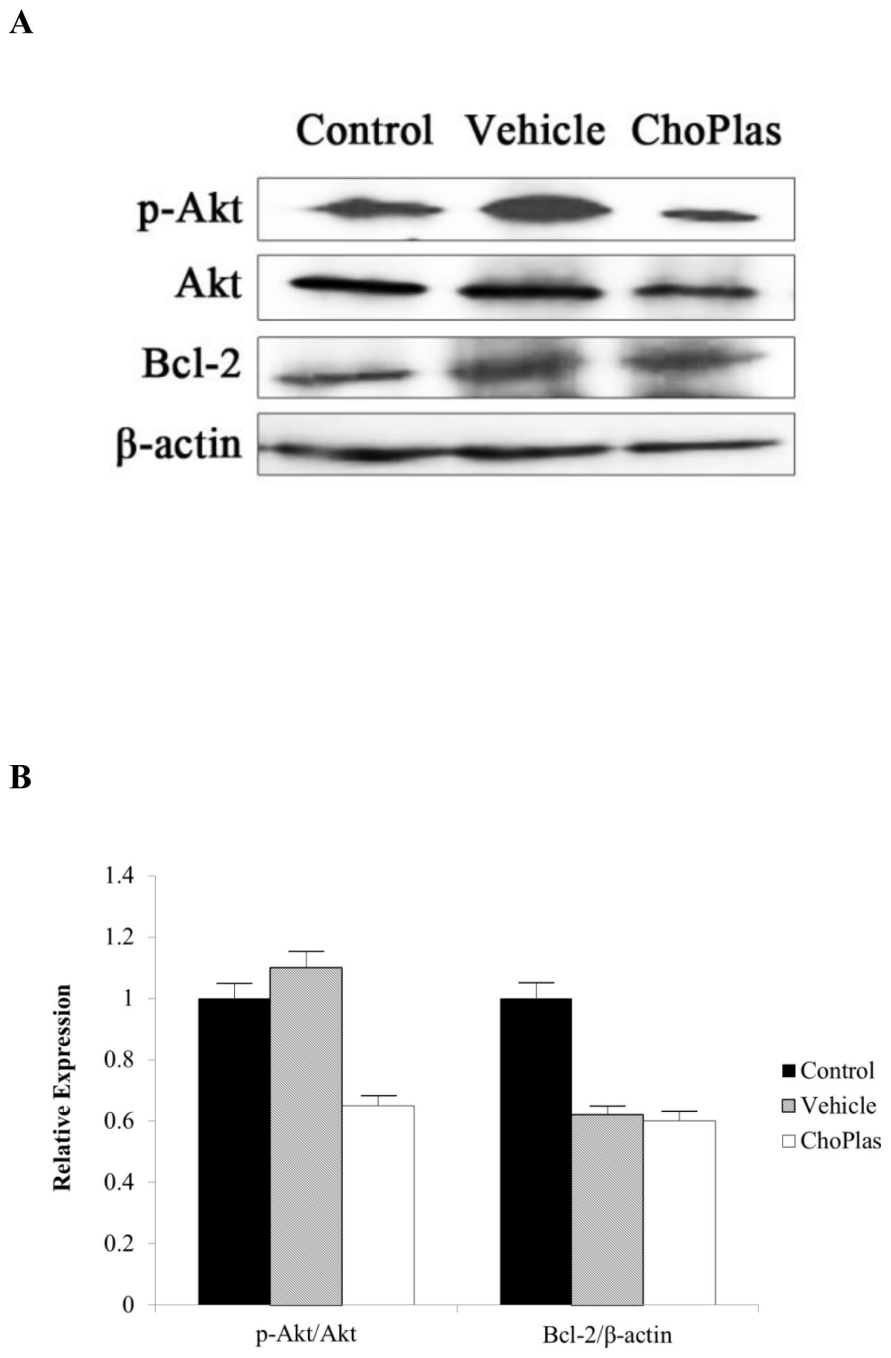
Effect of ChoPlas on Akt/PI3K pathway proteins. CBRH7919 cells were treated with 25 μmol/L of ChoPlas for 24 h and then harvested. (A)Total protein was extracted and then blotted with phosphor-Akt(Thr^308^), total Akt and Bcl-2 antibodies; (B) Graph depicting the semi-quantization of phosphor-Akt(Thr^308^), total Akt and Bcl-2 expression computed using Image J software. Data are presented as the mean±S.D (n=3).

### Caveolin-1 expression after ChoPlas treatment

Caveolin-1 has been shown to be involved in lipogenesis and the regulation of cellular proliferation [25]. Several studies indicate that specific environmental stimuli can increase caveoloin-1 expression [26,27]. To test the hypothesis that exogenous natural ChoPlas treatment can act as an environmental stimulus to alter the expression of caveolin-1, caveolin-1 expression in CBRH 7919 cells after ChoPlas treatment was examined. As shown in Figure 4, caveolin-1 levels increased significantly following ChoPlas treatment (25 μmol/L) for 24 h.

### Total Akt, pAkt and Bcl-2 expression following ChoPlas treatment

It is known that the phosphatidylinositol 3-kinase (PI3K)/Akt pathway is a major survival pathway in human cancers [28-32] and is regulated by caveolin-1[33,34]. Moreover, Akt, an important downstream target of the PI3K/Akt pathway plays a central role in anti-apoptosis through the phosphorylation and regulation of a variety of cell survival-related downstream targets[35].

The expression of total Akt and pAkt in CBRH7919 cells after ChoPlas treatment was detected. Figure 5 indicates that the expression of pAkt decreased significantly in ChoPlas-induced CRBH-7919 proliferation inhibition. Since Akt is a major mediator of cell survival through the direct inhibition of Bcl-2 family members, further apoptosis in ChoPlas-treated hepatoma cells was detected by measuring Bcl-2 protein expression. Figure 5 shows that Bcl-2 expression decreased significantly in ChoPlas-induced CRBH-7919 proliferation inhibition.

**Figure 6:**
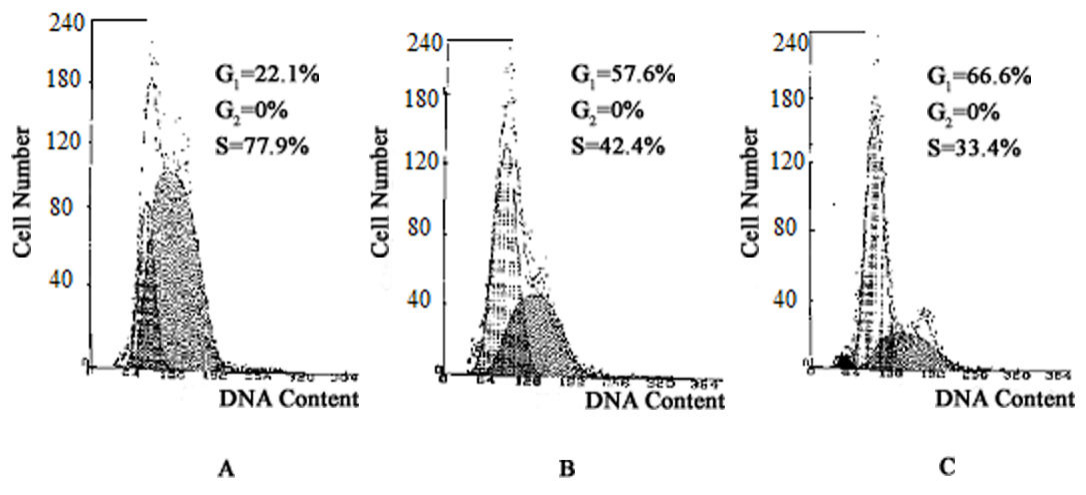
Effect of ChoPlas on the cell cycle of cultured CBRH-7919 cells. Cells were incubated with 25 μmol/L ChoPlas for 24 h. The cell cycle was analyzed by flow cytometry. A: Control. B: Vehicle . C: In the presence of ChoPlas.

### Cell cycle analysis by flow cytometry

Cell fate is determined not only by apoptosis but also by mitosis. Because the PI3K/Akt pathway regulates cell cycle progression indirectly, the impact of ChoPlas on the cell-cycle progression of CBRH-7919 cells was further assessed. Cells were treated with ChoPlas (25μm/L) for 24 h. Figure 6 shows that ChoPlas treatment resulted in a time dependent significant accumulation of cells in the G_1_ phase with a concomitant 14.1 % loss from the S phase. These results suggest that ChoPlas has an anti-proliferative effect on cells and possibly induces cell-cycle arrest at the G_1_/S phase transition (Figure 6A and C).

### Cyclin-dependent kinase (CDK) expression after ChoPlas treatment

**Figure 7:**
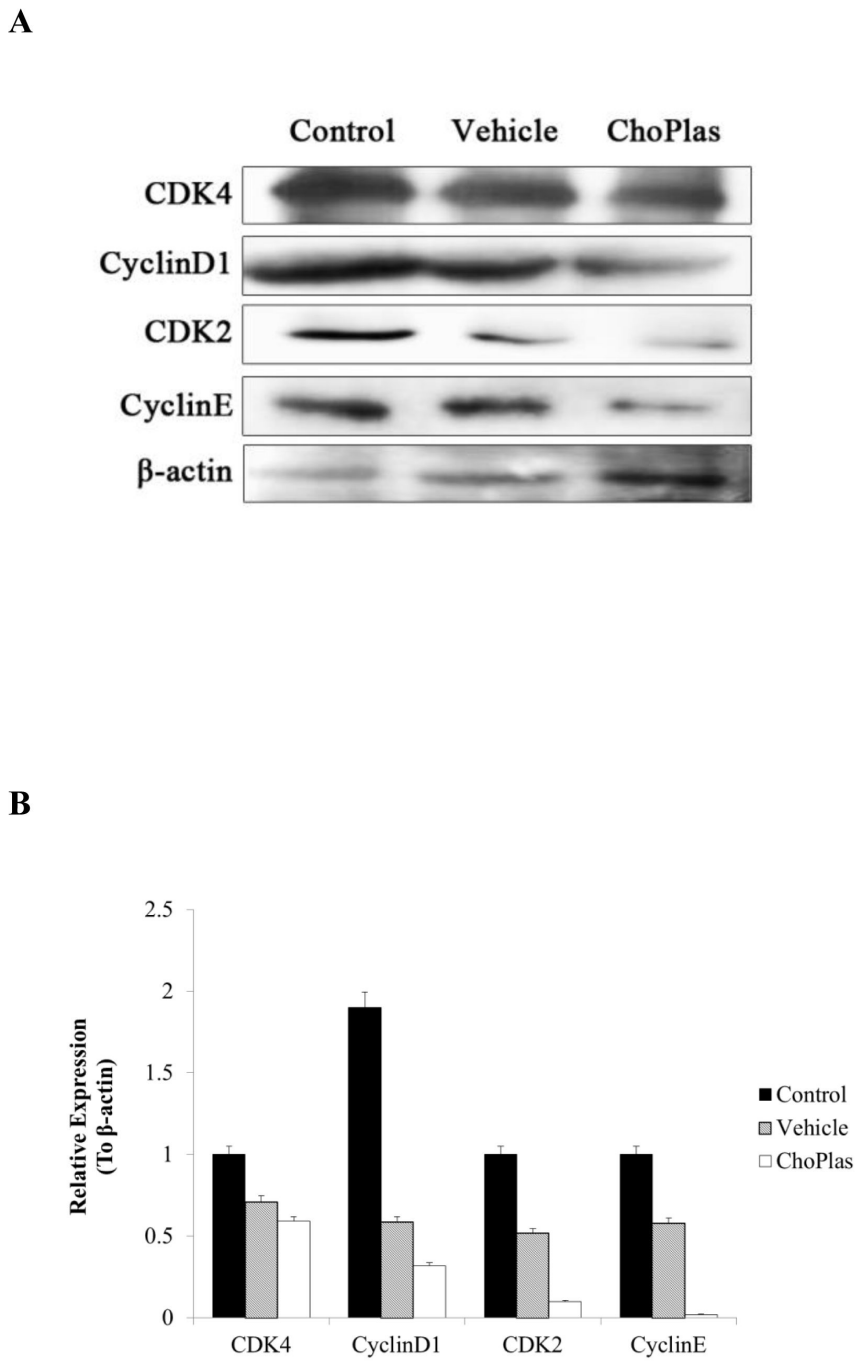
Cell cycle regulator protein expression following ChoPlas treatment. CBRH7919 cells were treated with 25 μmol/L ChoPlas for 24 h and then harvested. (A) Total protein was extracted and then blotted with Cyclin D1, CDK4, Cyclin E and CDK2 antibodies; (B) Graph depicting the semi-quantization of Cyclin D1, CDK4, Cyclin E and CDK2 expression computed using Image J software. Data are presented as the mean±S.D (n=3).

Based upon the results of the cell cycle assay, it appeared that essential cell cycle regulation factors could be involved in the inhibition caused by ChoPlas treatment. Therefore, essential factors in the G_1_ and G_1_/S progression, Cyclin D1/CDK4 and Cyclin E/CDK2, were chosen and their expression examined following ChoPlas treatment. As shown in Figure 7, ChoPlas treatment decreased CDK4，Cyclin E and CDK2 levels. The results indicate that CDK4 and CyclinE /CDK2 expression levels were attenuated by Caveolin-1 up-regulation and pAkt down-regulation.

## Discussion

Plasmalogens are major cellular structural and functional lipids that were originally found in myelin by Feulgen and Voit in 1924; their structure was deduced several years later [36]. They constituted 15-20% of total phospholipids in cell membranes and mainly distributed in heart, brain, kidney, lung and skeletal muscles whiles there are the lowest amounts in liver, which could be explained by their synthesis in liver [3,37] .The biological function of plasmalogens and their implication in diseases has remained elusive, particularly the function of exogenous natural liver –derived plasmalogens in human health. In the present work, an optimized HPTLC method for natural ChoPlas isolation and a HPLC/MS method of natural ChoPlas identification were successfully established.

Because plasmalogens are known to be involved in cancer and our primary research showed that exogenous natural phospholipids can inhibit rat hepatoma CBRH7919 cell proliferation (data not shown), we hypothesized that exogenous natural ChoPlas might have an effect on cancer cell proliferation. We found that ChoPlas can inhibit hepatoma cell proliferation with an optimal concentration and time of 25 μmol/L and 24 h. To further demonstrate that ChoPlas specifically inhibited cancer cell proliferation，human hepatoma HepG2 and SMMC 7721, human normal hepatic cells HL7702 as well as Chang’s live cells were used. These experiments further characterized the effects of ChoPlas treatment and provided a broader comparison of its effects on the growth of normal human hepatic cell lines and human hepatoma cell lines compared to rat hepatoma CBRH7919 cells. It suggested that liver-derived ChoPlas could inhibite the proliferation of hepatoma cells but not other normal hepatic cells.

Plasmalogens and Caceolin-1 are known to play important roles in cell membrane lipid homeostasis [12,13,38,39] and are both involved in cancer [15,19]. In the present study, it was shown that exogenous natural ChoPlas treatment activated Caveolin-1 expression in hepatoma CBRH7919 cells, indicating that exogenous ChoPlas-induced cell proliferation inhibition is dependent on caveolin-1 over-expression. This result is consistent with several studies, suggesting that exogenous stimuli-induced caveolin-1 over-expression can reduce cell proliferation [40,41]. However, the exact interaction between exogenous natural ChoPlas and Caveolin-1 is still not known and further work will be required to understand how exogenous natural ChoPlas interacts with Caveolin-1 within the cell membrane.

Recent studies indicate that the phosphatidylinositol 3-kinase (PI3K)/Akt(PI3K/Akt) signaling pathway is overactive in cancer and therefore presents a promising target for cancer therapy [42-44]. Moreover, it was reported that PI3K/Akt exists in caveolae[45] and is regulated by caveolin-1. Our data suggests that ChoPlas-induced caveolin-1 over-expression down-regulates PI3K/Akt phosphorylation downstream of Akt in hepatoma CBRH7919 cells. Akt is reported to play a critical role in anti-apoptotic pathways through the phosphorylation and regulation of a variety of cell survival-related downstream targets in prostate cancer [35]. Our results show that exogenous ChoPlas-induced caveolin-1 overexpression can reduce pAkt levels in hepatoma cells, consistent with two previous studies which found that down-regulation of the PI3K/Akt pathway in caveolin-1 overexpressing cells sensitizes them to apoptotic stimuli [46,47].

The present work also supports the previous proposal that signaling complexes exist within caveolae that could potentially respond to extracellular stimuli[48]. However, it is important to note that the influence of caveolin-1 overexpression on the PI3K/Akt signaling pathway and on Akt activity may also be dependent on stress conditions, cell type, or both. Shack et al. reported that elevated Akt activity appears to be important in sensitizing caveolin-1 overexpressing cells to arsenite-induced toxicity, indicating that caveolin-induced up-regulation of the PI3K/Akt signaling pathway appears to be a death signal in the presence of arsenite and that H_2_O_2_ sensitizes cells to environmental stress [33]. Park et al. reported that EGF-induced caveolin-1 overexpression can increase Akt phosphorylation levels in mouse embryonic stem cells [41]. The differences between our findings and those reported by Shack et al. and Park et al. can be accounted for by considering their use of normal cells (ES-E14TG2a, human cell lines 293 and HeLa) compared with the use of cancer cells (hepatoma CBRH7919) in our study.

Additionally, Akt promotes cell survival through the direct regulation of Bcl-2 family members (Bcl-2, Bcl-X(L) and Mcl-2) [49,50]. Previous studies have indicated that Bcl-2 is highly overexpressed in some cancers and enhances cancer cell survival [51,52]. Our results show that exogenous ChoPlas treatment can reduce the expression of Bcl-2, an indication that exogenous natural ChoPlas affects cancer cell proliferation by promoting cancer cell apoptosis.

Uncontrolled cell proliferation and abnormal cell cycle regulation are main characteristics of human cancers. In many studies, both caveolin-1 and Akt have been shown to regulate cell proliferation and the cell cycle through their direct or indirect action on different signaling pathway targets and cell cycle regulatory factors [53]. Eymin et al. reported that the disruption of the G_1_/S phase transition is a crucial event during lung carcinogenesis [54]. The results of our study indicate that exogenous natural ChoPlas can affect both G_1_ and G_1_/S phase transitions in the hepatoma cell cycle. CyclinD1/CDK4 and CyclinE/CDK2 are essential regulatory factors involved in the G_1_ and G_1_/S phase transitions of cell cycle progression [54,55]. CyclinD1/CDK4 is expressed in G_1_ and in late G_1_. CyclinE accumulates and associates with CDK2 to irreversibly initiate gene expression required in S phase (G_1_/S phase transition). This restriction point is crucial because it is here that cells proliferate independently of mitogenic stimuli in cancer[54].

**Figure 8:**
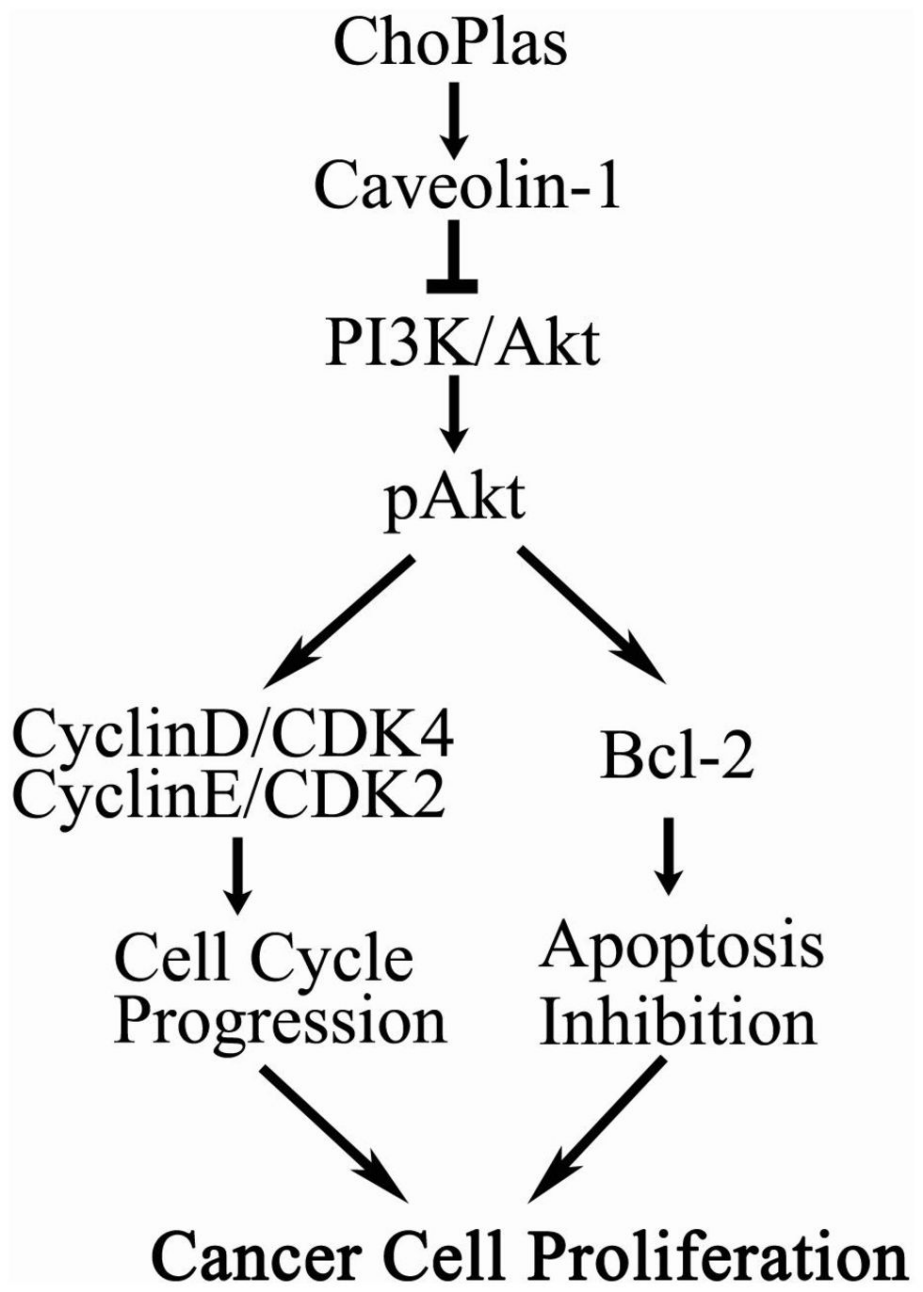
Hypothesized model for the proliferation inhibition mechanism of ChoPlas on hepatoma cells. Exogenous natural ChoPlas activates caveolin-1, affecting PI3K/Akt pathway signals and decreasing cell cycle regulatory proteins and Bcl-2 expression.

Though further work is needed to identify the targets involved in ChoPlas-induced alterations of cell cycle regulation, our data show that CDK4, CyclinE and CDK2 levels were attenuated by exogenous ChoPlas treatment, suggesting that exogenous ChoPlas inhibits hepatoma cell proliferation through its action on cell cycle regulatory factors. Furthermore, natural ChoPlas inhibited hepatoma cell proliferation via the PI3K/Akt pathway and was dependent on caveolin-1 activation (Figure 8), thus presenting natural ChoPlas as a potential cancer drug candidate.

## Supporting Information

Figure S1 **Inhibition rate with treatment of ChoPlas by MTT test (24h and 48h).** CBRH-7919, HepG2, SMMC7721, HL7702 and Chang’s liver cells (1×104 cells/well) were seeded in a 96-well plate with a medium supplemented with 10% FBS and incubated for 24 h, respectively. Cells were then treated with 25μmol/L of ChoPlas in 20mmol/L ethanol for 24h and 48h. And then viable cells were stained with MTT and resulting formazan crystals were dissolved with DMSO. The absorbance at 490nm was measured with a multiscan plate reader. Data are presented as the mean±S.D. n=3).
(TIF)

## References

[1] Braverman NE, Moser AB ( 2012 ) Functions of plasmalogen lipids in health and disease. Biochim Biophys Acta 1822: 1422-1452. PubMed: 22627108. 10.1016/j.bbadis.2012.05.008 22627108

[2] Brites P, Waterham HR, Wanders RJ ( 2004 ) Functions and biosynthesis of plasmalogens in health and disease. Biochim Biophys Acta 1636(2-3): 219-231. doi:10.1016/j.bbalip.2003.12.010. PubMed: 15164770. 15164770

[3] Vance JE ( 1990 ) Lipoproteins secreted by cultured rat hepatocytes contain the antioxidant 1-alk-1-enyl-2-acylglycerophosphoethanolamine. Biochim Biophys Acta 1045 : 128-134. doi:10.1016/0005-2760(90)90141-J. PubMed: 2116174. 2116174

[4] Heymans HS, Schutgens RB, Tan R, van den Bosch H, Borst P ( 1983 ) Severe plasmalogen deficiency in tissues of infants without peroxisomes (Zellweger syndrome). Nature 306(5938): 69-70. doi:10.1038/306069a0. PubMed: 6633659. 6633659

[5] Wood PL, Khan MA, Smith T, Ehrmantraut G, Jin W et al. ( 2011 ) In vitro and in vivo plasmalogen replacement evaluations in rhizomelic chrondrodysplasia punctata and Pelizaeus-Merzbacher disease using PPI-1011, an ether lipid plasmalogen precursor. Lipids Health Dis 10: 182. doi:10.1186/1476-511X-10-182. PubMed: 22008564. 22008564 PMC3238230

[6] Farooqui AA, Rapoport SI, Horrocks LA ( 1997 ) Membrane phospholipid alterations in Alzheimer's disease: deficiency of ethanolamine plasmalogens. Neurochem Res 22(4): 523-527. doi:10.1023/A:1027380331807. PubMed: 9130265. 9130265

[7] Igarashi M, Ma K, Gao F, Kim HW, Rapoport SI et al. ( 2011 ) Disturbed choline plasmalogen and phospholipid fatty acid concentrations in Alzheimer's disease prefrontal cortex. J Alzheimers Dis 24(3): 507-517. PubMed: 21297269. 21297269 10.3233/JAD-2011-101608 PMC3175096

[8] Schedin S, Sindelar PJ, Pentchev P, Brunk U, Dallner G ( 1997 ) Peroxisomal impairment in Niemann-Pick type C disease. J Biol Chem 272(10): 6245-6251. doi:10.1074/jbc.272.10.6245. PubMed: 9045641. 9045641

[9] Murphy EJ, Schapiro MB, Rapoport SI, Shetty HU ( 2000 ) Phospholipid composition and levels are altered in Down syndrome brain. Brain Res 867(1-2): 9-18. doi:10.1016/S0006-8993(00)02205-8. PubMed: 10837793. 10837793

[10] Kohlschütter A, Schade B, Blömer B, Hübner C ( 1993 ) Low erythrocyte plasmalogen and plasma docosahexaenoic acid (DHA) in juvenile neuronal ceroid-lipofuscinosis (JNCL). J Inherit Metab Dis 16(2): 299-304. doi:10.1007/BF00710270. PubMed: 8411986. 8411986

[11] Schaefer EJ, Robins SJ, Patton GM, Sandberg MA, Weigel-DiFranco CA et al. ( 1995 ) Red blood cell membrane phosphatidylethanolamine fatty acid content in various forms of retinitis pigmentosa. J Lipid Res 36(7): 1427-1433. PubMed: 7595066. 7595066

[12] Zoeller RA, Grazia TJ, LaCamera P, Park J, Gaposchkin DP et al. ( 2002 ) Increasing plasmalogen levels protects human endothelial cells during hypoxia. Am J Physiol Heart Circ Physiol 283(2): H671-H679. PubMed: 12124215. 12124215 10.1152/ajpheart.00524.2001

[13] Mandel H, Sharf R, Berant M, Wanders RJ, Vreken P et al. ( 1998 ) Plasmalogen phospholipids are involved in HDL-mediated cholesterol efflux: insights from investigations with plasmalogen-deficient cells. Biochem Biophys Res Commun 250(2): 369-373. doi:10.1006/bbrc.1998.9321. PubMed: 9753636. 9753636

[14] Mankidy R, Ahiahonu PW, Ma H, Jayasinghe D, Ritchie SA et al. ( 2010 ) Membrane plasmalogen composition and cellular cholesterol regulation: a structure activity study. Lipids Health Dis 9: 62. doi:10.1186/1476-511X-9-62. PubMed: 20546600. 20546600 PMC2902472

[15] Wood PL, Khan MA, Smith T, Goodenowe DB ( 2011 ) Cellular diamine levels in cancer chemoprevention: modulation by ibuprofen and membrane plasmalogens. Lipids Health Dis 10: 214. doi:10.1186/1476-511X-10-214. PubMed: 22087745. 22087745 PMC3231815

[16] Williams TM , Lisanti MP ( 2005 ) Caveolin-1 in oncogenic transformation, cancer, and metastasis. Am J Physiol Cell Physiol 288: 494-506. PubMed: 15692148. 10.1152/ajpcell.00458.2004 15692148

[17] Wang X, Feng S, Zhang H, Wang Y, Cui YY et al. ( 2011 ) RNA inference-mediated caveolin-1 gene silencing decrease estrogen receptor alpha (ERα) signaling in human mammary epithelial cells. Mol Biol Rep 2: 761-768. 10.1007/s11033-010-0164-5 20411337

[18] Ren G, Liu Y, Wand XM, Zhao CH, Zou W ( 2008 ) Role of caveolin-1 down-regulation by iRNA in human hepatocyte proliferation.Chin J Hepatol 16: 379-382 18510854

[19] Gargalovic P, Dory L ( 2003 ) Caveolins and macrophage lipid metabolism. J Lipid Res 44(1): 11-21. doi:10.1194/jlr.R200005-JLR200. PubMed: 12518018. 12518018

[20] Burgermeister E, Xing X, Röcken C, Juhasz M, Chen J et al. ( 2007 ) Differential expression and function of caveolin-1 in human gastric cancer progression. Cancer Res 67(18): 8519-8526. doi:10.1158/0008-5472.CAN-07-1125. PubMed: 17875691. 17875691

[21] Folch J, Lee MB, Sloane-Standey GH ( 1957 ) A simple method for the isolation and purification of total lipids from animal tissues. J Biol Chem 226: 497-509. PubMed: 13428781. 13428781

[22] Wang L, Liu CP, Jia D, Zou W ( 2006 ) Separation and purification of phosphatidylcholine in swine liver and its inhibition effect on proliferation of rat hepatoma cells. Chinese Journal of Chromatography 24(3): 271-274 16929847

[23] Cui Z, Vance JE, Chen MH, Voelker DR, Vance DE ( 1993 ) Cloning and expression of a novel phosphatidylethanolamine N-methyltransferase. A specific biochemical and cytological marker for a unique membrane fraction in rat liver. J Biol Chem 268(22): 16655-16663. PubMed: 8344945. 8344945

[24] Zou W, Li ZY, Li YL, Ma KL, Tsui ZC ( 2002 ) Overexpression of PEMT2 downregulates the PI3K/Akt signaling pathway in rat hepatoma cells. Biochim Biophys Acta 1581(1-2): 49-56. doi:10.1016/S1388-1981(02)00120-8. PubMed: 11960751. 11960751

[25] Li Y, Zou W, Yan Q, Xu Y, Xia Q et al. ( 2009 ) Over-expression of pemt2 into rat hepatoma cells contributes to the mitochondrial apoptotic pathway. IUBMB Life 61(8): 846-852. doi:10.1002/iub.222. PubMed: 19517528. 19517528

[26] Frank PG, Lisanti MP ( 2007 ) Caveolin-1 and liver regeneration: role in proliferation and lipogenesis. Cell Cycle 6(2): 115-116. doi:10.4161/cc.6.2.3722. PubMed: 17314510. 17314510

[27] Esser S, Wolburg K, Wolburg H, Breier G, Kurzchalia T et al. ( 1998 ) Vascular endothelial growth factor induces endothelial fenestrations in vitro. J Cell Biol 140(4): 947-959. doi:10.1083/jcb.140.4.947. PubMed: 9472045. 9472045 PMC2141756

[28] Demou ZN ( 2010 ) Gene expression profiles in 3D tumor analogs indicate compressive strain differentially enhances metastatic potential. Ann Biomed Eng 38(11): 3509-3520. doi:10.1007/s10439-010-0097-0. PubMed: 20559731. 20559731

[29] Chen YL, Law PY, Loh HH ( 2005 ) Inhibition of PI3K/Akt signaling: an emerging paradigm for targeted cancer therapy. Curr Med Chem Anti Cancer Agents 5(6): 575-589. doi:10.2174/156801105774574649. PubMed: 16305480. 16305480

[30] Marinov M, Fischer B, Arcaro A ( 2007 ) Targeting mTOR signaling in lung cancer. Crit Rev Oncol/Hematol 63(2): 172-182. doi:10.1016/j.critrevonc.2007.04.002. PubMed: 17540577. 17540577

[31] Ghayad SE, Cohen PA ( 2010 ) Inhibitors of the PI3K/Akt/mTOR pathway: new hope for breast cancer patients. Recent Pat Anticancer Drugs Discov 5(1): 29-57. doi:10.2174/157489210789702208. PubMed: 19751211. 19751211

[32] Falasca M ( 2010 ) PI3K/Akt signalling pathway specific inhibitors: a novel strategy to sensitize cancer cells to anti-cancer drugs. Curr Pharm Des 16(12): 1410-1416. doi:10.2174/138161210791033950. PubMed: 20166984. 20166984

[33] Zhou Q, Lui VW, Yeo W ( 2011 ) Targeting the PI3K/Akt/mTOR pathway in hepatocellular carcinoma. Future Oncol 7(10): 1149-1167. doi:10.2217/fon.11.95. PubMed: 21992728. 21992728

[34] Shack S, Wang XT, Kokkonen GC, Gorospe M, Longo DL et al. ( 2003 ) Caveolin-induced activation of the phosphatidylinositol 3-kinase/Akt pathway increases arsenite cytotoxicity. Mol Cell Biol 23(7): 2407-2414. doi:10.1128/MCB.23.7.2407-2414.2003. PubMed: 12640124. 12640124 PMC150728

[35] Park JH, Ryu JM, Han HJ ( 2011 ) Involvement of caveolin-1 in fibronectin-induced mouse embryonic stem cell proliferation: role of FAK, RhoA, PI3K/Akt, and ERK 1/2 pathways. J Cell Physiol 226(1): 267-275. doi:10.1002/jcp.22338. PubMed: 20658539. 20658539

[36] Rapport MM ( 1984 ) The discovery of plasmalogen structure. J Lipid Res 25(13) (1522-1527) 6397557

[37] Braverman NE, Moser AB ( 2012 ) Functions of plasmalogen lipids in health and disease. Biochim Biophys Acta 1822: 1442-1452. doi:10.1016/j.bbadis.2012.05.008. PubMed: 22627108. 22627108

[38] Smart EJ, Ying Ys, Donzell WC, Anderson RG ( 1996 ) A role for caveolin in transport of cholesterol from endoplasmic reticulum to plasma membrane. J Biol Chem 271(46): 29427-29435. doi:10.1074/jbc.271.46.29427. PubMed: 8910609. 8910609

[39] Liu P, Li WP, Machleidt T, Anderson RG ( 1999 ) Identification of caveolin-1 in lipoprotein particles secreted by exocrine cells. Nat Cell Biol 1(6): 369-375. doi:10.1038/14067. PubMed: 10559965. 10559965

[40] Park WY, Park JS, Cho KA, Kim DI, Ko YG et al. ( 2000 ) Up-regulation of caveolin attenuates epidermal growth factor signaling in senescent cells. J Biol Chem 275(27): 20847-20852. doi:10.1074/jbc.M908162199. PubMed: 10781609. 10781609

[41] Park JH, Han HJ ( 2009 ) Caveolin-1 plays important role in EGF-induced migration and proliferation of mouse embryonic stem cells: involvement of PI3K/Akt and ERK. Am J Physiol Cell Physiol 297(4): C935-C944. doi:10.1152/ajpcell.00121.2009. PubMed: 19625610. 19625610

[42] Morgensztern D, McLeod HL ( 2005 ) PI3K/Akt/mTOR pathway as a target for cancer therapy. Anti Cancer Drugs 16(8): 797-803. doi:10.1097/01.cad.0000173476.67239.3b. PubMed: 16096426. 16096426

[43] Yap TA, Garrett MD, Walton MI, Raynaud F, de Bono JS et al. ( 2008 ) Targeting the PI3K-AKT-mTOR pathway: progress, pitfalls, and promises. Curr Opin Pharmacol 8(4): 393-412. doi:10.1016/j.coph.2008.08.004. PubMed: 18721898. 18721898

[44] LoPiccolo J, Blumenthal GM, Bernstein WB, Dennis PA ( 2008 ) Targeting the PI3K/Akt/mTOR pathway: effective combinations and clinical considerations. Drug Resist Update 11(1-2): 32-50. doi:10.1016/j.drup.2007.11.003. PubMed: 18166498. PMC2442829 18166498

[45] Anderson RG ( 1993 ) Caveolae: where incoming and outgoing messengers meet. Proc Natl Acad Sci U S A 90(23): 10909-10913. doi:10.1073/pnas.90.23.10909. PubMed: 8248193. 8248193 PMC47891

[46] Liu J, Lee P, Galbiati F, Kitsis RN, Lisanti MP ( 2001) Caveolin-1 expression sensitizes fibroblastic and epithelial cells to apoptotic stimulation. Am J Physiol Cell Physiol 280(4): C823-C835. PubMed: 11245599. 11245599 10.1152/ajpcell.2001.280.4.C823

[47] Zundel W, Swiersz LM, Giaccia A ( 2000 ) Caveolin 1-mediated regulation of receptor tyrosine kinase-associated phosphatidylinositol 3-kinase activity by ceramide. Mol Cell Biol 20(5): 1507-1514. doi:10.1128/MCB.20.5.1507-1514.2000. PubMed: 10669728. 10669728 PMC85322

[48] Lisanti MP, Scherer PE, Tang Z, Sargiacomo M ( 1994 ) Caveolae, caveolin and caveolin-rich membrane domains: a signalling hypothesis. Trends Cell Biol 4(7): 231-235. doi:10.1016/0962-8924(94)90114-7. PubMed: 14731661. 14731661

[49] Downward J ( 2004 ) PI 3-kinase, Akt and cell survival. Semin Cell Dev Biol 15(2): 177-182. doi:10.1016/j.semcdb.2004.01.002. PubMed: 15209377. 15209377

[50] Maddika S, Ande SR, Panigrahi S, Paranjothy T, Weglarczyk K et al. ( 2007 ) Cell survival, cell death and cell cycle pathways are interconnected: implications for cancer therapy. Drug Resist Update 10(1-2): 13-29. doi:10.1016/j.drup.2007.01.003. PubMed: 17303468. 17303468

[51] Nadler Y, Camp RL, Giltnane JM, Moeder C, Rimm DL et al. ( 2008 ) Expression patterns and prognostic value of Bag-1 and Bcl-2 in breast cancer. Breast Cancer Res 10(2): R35. doi:10.1186/bcr1998. PubMed: 18430249. 18430249 PMC2397537

[52] Straten Pt, Andersen MH ( 2010 ) The anti-apoptotic members of the Bcl-2 family are attractive tumor-associated antigens. Oncotarget 1(4): 239-245. PubMed: 21304176. 21304176 10.18632/oncotarget.134 PMC3248102

[53] Guruswamy S, Rao CV ( 2009 ) Synergistic effects of lovastatin and celecoxib on caveolin-1 and its down-stream signaling molecules: Implications for colon cancer prevention. Int J Oncol 35(5): 1037-1043. PubMed: 19787257. 19787257 10.3892/ijo_00000418

[54] Eymin B, Gazzeri S ( 2010 ) Role of cell cycle regulators in lung carcinogenesis. Cell Adh Migr 4(1): 114-123. doi:10.4161/cam.4.1.10977. PubMed: 20139697. 20139697 PMC2852568

[55] Lange C, Calegari F ( 2010) Cdks and cyclins link G1 length and differentiation of embryonic, neural and hematopoietic stem cells. Cell Cycle 9(10): 1893-1900. doi:10.4161/cc.9.10.11598. PubMed: 20436288. 20436288

